# The Assessment of Retinal Image Quality Using a Non-Mydriatic Fundus Camera in a Teleophthalmologic Platform

**DOI:** 10.3390/diagnostics14141543

**Published:** 2024-07-17

**Authors:** Tsung-Yueh Chan, Jen-Hung Wang, Nancy Chen, Cheng-Jen Chiu

**Affiliations:** 1 Department of Ophthalmology, Hualien Tzu Chi Hospital, Buddhist Tzu Chi Medical Foundation, Hualien 970, Taiwan; willi81404@gmail.com; 2 Department of Medical Research, Hualien Tzu Chi Hospital, Buddhist Tzu Chi Medical Foundation, Hualien 970, Taiwan; jenhungwang2011@gmail.com; 3 Department of Ophthalmology and Visual Science, Tzu Chi University, Hualien 970, Taiwan

**Keywords:** teleophthalmology, non-mydriatic fundus camera, retinal image quality, remote screening, ocular diseases, diabetes mellitus, cataracts, pseudophakia

## Abstract

This study assesses the quality of retinal images captured using a non-mydriatic fundus camera within a teleophthalmologic platform in Taiwan. The objective was to evaluate the effectiveness of non-mydriatic fundus cameras for remote retinal screening and identify factors impacting image quality. From June 2020 to August 2022, 629 patients from five rural infirmaries underwent ophthalmic examinations, with fundus images captured without pupil dilation. These images were reviewed by senior ophthalmologists and graded based on quality. The results indicated that approximately 70% of images were of satisfactory diagnostic quality. Risk factors for poor image quality included older age, the presence of cataracts, pseudophakia, and diabetes mellitus. This study demonstrates the feasibility of using non-mydriatic fundus cameras for teleophthalmology, highlighting the importance of identifying and addressing factors that affect image quality to enhance diagnostic accuracy in remote settings.

## 1. Introduction

Telemedicine, or remote healthcare, has become an essential solution for overcoming geographical barriers and the uneven distribution of resources [1]. Teleophthalmology, a branch of telemedicine, leverages electronic data and medical technology through digital devices and telecommunications to enhance health outcomes by providing quality eye care to individuals in remote areas or with limited resources [2]. It transcends geographical limitations for eye examinations and narrows the disparity in ophthalmic care between rural and urban areas. With advances in technology and imaging tools, digital retinal photography has become crucial in advancing ophthalmic diagnostics. Digital images taken remotely can be electronically transmitted to an image reading center staffed by expert clinicians who perform disease screening and diagnosis. Those suspected of having significant ocular disorders can then be referred to an ophthalmologist for a comprehensive evaluation and treatment [3,4].

While stereoscopic mydriatic seven-field 30-degree photography is widely regarded as the gold standard for detecting and classifying diabetic retinopathy due to its high sensitivity and specificity, as defined by the Early Treatment Diabetic Retinopathy Study (ETDRS) [5], images obtained by non-mydriatic fundus cameras can also effectively serve as a screening tool. This alternative method can provide favorable technical outcomes and help identify patients with retinal diseases [5,6,7]. Additionally, it eliminates the need for pharmacologic dilation in teleophthalmology services. In remote clinical settings, administering pharmacologic pupil dilation agent without first confirming the absence of narrow anterior chamber angles by an ophthalmologist may lead to post-dilation intraocular pressure elevation [8] and even an acute angle-closure glaucoma attack, which is an emergent, vision-threatening condition in ophthalmology.

The aim of this study was to implement and evaluate a teleophthalmology screening system using non-mydriatic fundus cameras. We analyzed the distribution of ocular diseases, assessed the quality of the non-mydriatic fundus images, and simultaneously established concordance of the diagnosis among the referrals. Additionally, we systematically evaluated the risk factors associated with poor image quality during our study.

## 2. Materials and Methods

### 2.1. Participants and the Setting

This prospective study collected ophthalmic telemedicine data from June 2020 to August 2022. It was conducted in accordance with the Declaration of Helsinki and received approval from the Institutional Review Board of Hualien Tzu Chi General Hospital (IRB109-198-B, 1 October 2020). Since 2020, the ophthalmology department at Tzu Chi General Hospital has been running a large-scale teleophthalmology program in collaboration with five rural infirmaries in Taitung County: Chishang, Haiduan, Guanshan, Luye, and Yanping. All patients who received teleophthalmology services at these infirmaries were included in this study. The details of the operation of the teleophthalmology module were described in our previous study [9].

### 2.2. Ophthalmic Examination, Image Acquisition, and Data Transmission

After registering patients from local infirmaries, we collected detailed demographic, general health, and eye condition data. The patients then underwent comprehensive ophthalmic examinations, which included measuring visual acuity using conventional Snellen charts, intraocular pressure, and taking external eye photographs along with a digital slit lamp image. The fundus photographs were captured using a non-mydriatic fundus camera (MiiS, Horus 5 Mp Auto focus Digital Fundus Camera DSC200, with an image resolution of 2560 × 1920 pixels) without pupillary dilation. The infirmary staff, who had undergone three months of training, performed these procedures to ensure a standardized process and consistent quality across different infirmaries.

The ocular examination results were transferred, via a continuous 80 MHz bandwidth 5G network, to the videoconferencing platform along with the images acquired using a digital slit lamp and fundus camera. Next, the general practitioner from the infirmary conducted a synchronous video consultation with the ophthalmologist, during which the data were input into the electronic medical records (EMR) and stored in the medical center terminal.

### 2.3. Clinical Diagnosis and Referral

**Figure 1:**
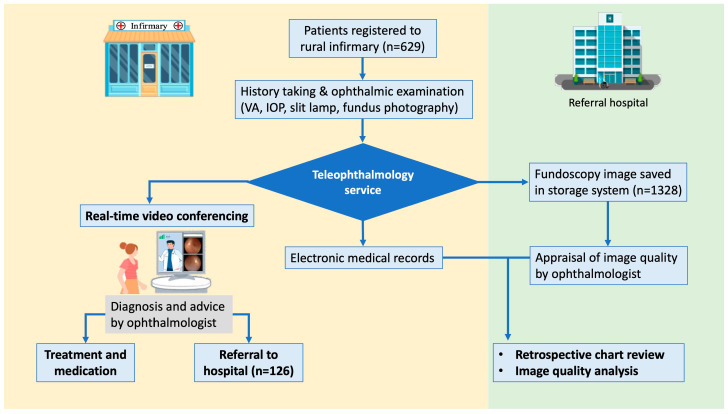
Flowchart illustrating the operation of our teleophthalmology service.

The primary physician at the infirmary conducted a real-time video consultation with an ophthalmologist from the medical center. Images from the digital slit lamp and non-mydriatic fundus camera were uploaded to the video consultation platform. The ophthalmologist made diagnoses and suggested treatments based on the patient’s real-time interview and the infirmary’s preliminary data and images (Figure 1). The diagnosis for each patient was recorded using codes from the International Classification of Diseases (ICD)-10. Patients were referred to a medical center for further evaluation or management if indicated.

### 2.4. Assessment of Diagnostic Agreement and Evaluation of Fundus Photographs

**Figure 2:**
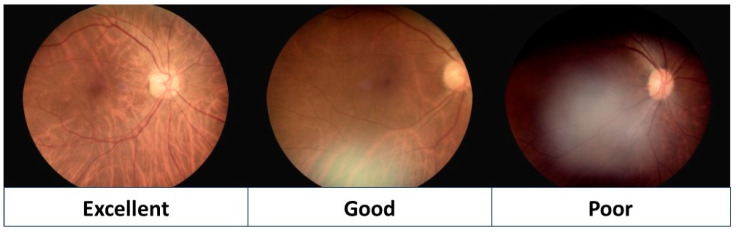
Image quality of non-mydriatic fundoscopy. The image quality was graded as “excellent” if blurred areas covered less than 25% of the fundus field; “good” if the blurred areas were between 25% and 50% of the fundus field; “poor” if more than 50% was blurred or obscured, or if main structures (the disc, macula, and vessels) were out of focus.

The concordance of diagnoses for referred patients was analyzed by comparing their ophthalmic records and ICD-10 codes from a teleophthalmology setting, with the diagnoses made at the hospital. Fundus photographs, taken using non-mydriatic fundoscopy, were organized and displayed on computer screens for quality assessment. Two masked, board-certified senior ophthalmologists (CJ/NC) independently reviewed the fundoscopy images and meticulously evaluated their quality. The quality of the images was graded as follows: “excellent” if blurred areas constituted less than 25% of the fundus field and the structures of the disc, macula, and vessels were clearly visible; “good” if the blurred areas covered more than 25% but less than 50% of the fundus field, yet still allowed clear identification of the main structures; “poor” if over 50% of the fundus field was obscured or blurred, or when the main structures were out of focus (Figure 2).

### 2.5. Statistical Analysis

Statistical analysis was conducted using SPSS software version 17.0 (SPSS Inc., Chicago, IL, USA). The baseline characteristics of the participants were presented as frequencies, proportions, or means ± standard deviations, based on the nature of each variable. An independent Student’s *t*-test was used to compare the means of continuous variables across different groups. The chi-squared test or Fisher’s exact test was employed to assess the distribution patterns of image quality and the relationships between two categorical variables. Differences were considered statistically significant at *p* < 0.05. The Kappa coefficient was adopted to evaluate the interrater reliability. Both simple and multiple logistic regression models were used to simultaneously analyze the association between poor image quality and various risk factors. Crude and adjusted odds ratios (ORs), along with 95% confidence intervals, were calculated.

## 3. Results

### 3.1. Participant Demographics and Characteristics

Our teleophthalmology service provided video consultations to 629 individuals in the area. There were 210 males and 419 females, resulting in a male-to-female ratio of 1:2. The mean age of participants was 64.40 ± 16.10 years. Approximately 70% of participants were more than 60 years of age. Gender distribution did not vary significantly across different age groups. A total of 126 patients (20.0%) were referred to an ophthalmology medical center for further management (Table 1).

### 3.2. Distribution of Ocular Diseases

**Figure 3:**
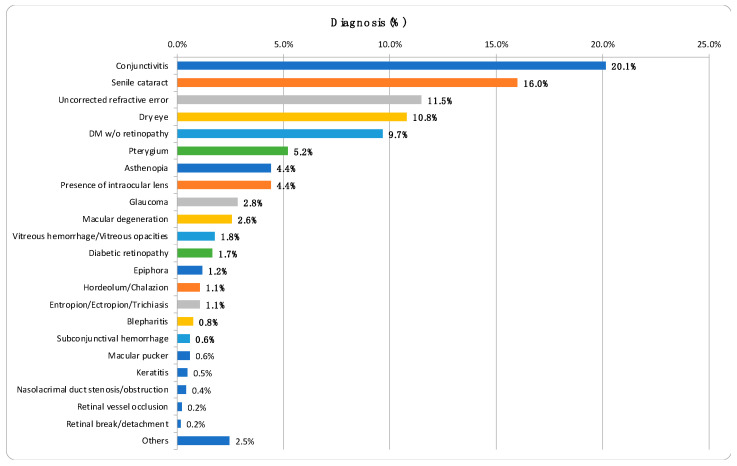
Comparison of the frequency of various ocular conditions.

The analysis of ICD-10 codes in the medical records revealed that chronic conjunctivitis was the most common eye condition among the patients, accounting for 20.1% of cases. This was followed by senile cataract at 16.0%, uncorrected refractive errors at 11.4%, and dry eye syndrome at 10.8%. There were a total of 211 visits (11.3%) for diabetic retinopathy screening, during which 180 patients (9.7%) showed no signs of the condition and 31 patients (1.7%) were diagnosed with diabetic retinopathy (Figure 3).

### 3.3. Evaluation of Fundoscopy Image Quality

**Figure 4:**
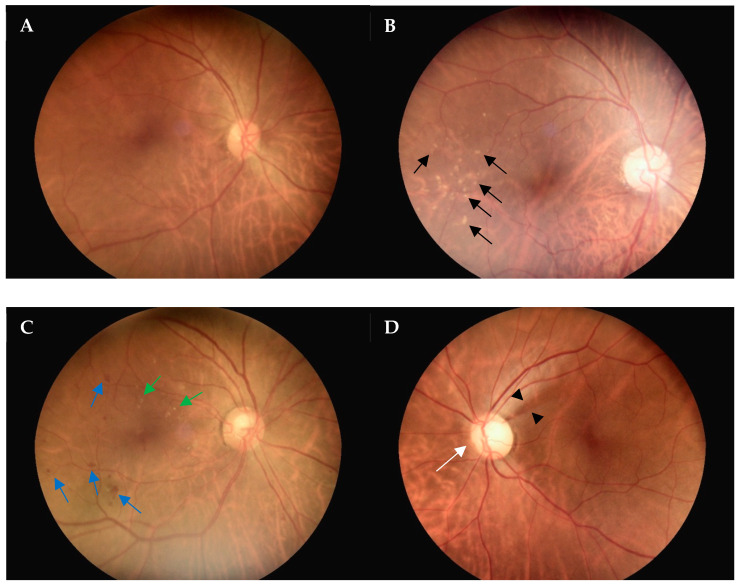
Examples of non-mydriatic fundus camera-captured photographs. (**A**), Fundus photograph demonstrates normal retina and optic disc. (**B**), Fundus photograph demonstrates the presence of a few drusen (black arrows), which is consistent with dry AMD. (**C**), Fundus photograph demonstrates the presence of blot hemorrhages (blue arrows) and hard exudates (green arrows), which is consistent with diabetic retinopathy. (**D**), Fundus photograph demonstrates enlargement of the optic disc cupping (white arrow) and retinal nerve fiber bundle defect (arrowheads), which is a sign of glaucoma. AMD, age-related macular degeneration.

The non-mydriatic digital fundus camera was used to capture fundus images of all patients without requiring pupil dilation. Each fundus color photograph was carefully evaluated for image quality to identify any subtle or obvious abnormalities. To assess the interrater reliability, we conducted an analysis and found that the Kappa coefficient was 0.65. Figure 4 displays fundus color photographs, illustrating a normal fundus (A), drusen (B), diabetic retinopathy (C), and enlarged cupping with nerve fiber layer loss (D), with all captured using a non-mydriatic fundus camera. A total of 1328 non-mydriatic fundoscopy images were obtained in this study. Of these images, 545 (41.0%) were graded as “excellent” and 374 (28.2%) as “good”, indicating that approximately 70% of the photos taken without pupil dilation were of a satisfactory quality for diagnostic purposes.

### 3.4. Diagnostic Concordance of the Referrals

One hundred and twenty-six patients were referred (Table 1). Among the patients referred, eighty-one (64.3%) underwent a second fundoscopy. Among them, sixty-three (77.8%) had a concordant diagnosis, while eighteen (22.2%) had a different or an additional diagnosis. Those additional diagnoses include macular pucker, diabetic macular edema, glaucoma, retinal break, and retinal lattice, which were detected subsequently by dilate fundoscopy, optical coherent tomography (OCT), or visual field test. The most frequently encountered reason for different diagnoses was image qualities, followed by equipment limitations.

### 3.5. Risk Factors Associated with Poor-Quality Fundoscopy Images

Of the fundoscopy images taken using the non-mydriatic digital fundus camera, 409 images (30.8%) were graded as “poor”. Univariate and multivariate repeated measurement analyses, along with multiple logistic regression models, were conducted to identify factors associated with poor image quality. The adjusted odds ratio results indicated that risk factors include cataracts, intraocular lens implants, diabetes mellitus, and older age (*p* < 0.001). However, gender and the location where the photos were taken (different infirmaries) did not significantly affect image quality (Table 2).

## 4. Discussion

In our public health initiative, we launched a synchronous teleophthalmology service that effectively screened a significant number of patients in rural eastern Taiwan. Our findings revealed that digital imaging is useful for diagnosing ocular diseases and screening for retinal conditions in remote populations. The most frequent diagnoses in our teleophthalmology practice included mild conditions such as conjunctivitis (20.1%), age-related cataract (16.0%), refractive errors (11.4%), and dry eye syndrome (10.8%). Additionally, approximately 9.8% of patients were diagnosed with retinal diseases. In a similar teleophthalmology service using real-time video consultations in rural Western Australia, the most common diagnosis was cataract (45.8%), followed by glaucoma (11.9%) and retinal disorders (10.1%) [10]. Similarly, to our findings, cataracts are a prevalent diagnosis in teleophthalmology services in rural settings. One of our goals was to identify individuals with treatable eye conditions and facilitate referrals to specialized ophthalmology clinics or hospitals for advanced care. In our study, 126 (20.3%) patients were referred for further evaluation at a tertiary care hospital.

Non-mydriatic fundus photography is a valuable clinical research tool for screening major ocular diseases, including age-related macular degeneration, glaucoma, and diabetic retinopathy [11,12]. Yeh’s study showed that the accuracy of diagnosing referable retinopathy with a non-mydriatic fundus camera is comparable to that of indirect fundoscopy after pupil dilation in real-world settings. The intergrader agreement on diagnosing referral-warranted retinopathy from fundus images was high, with most images graded as acceptable or of ideal quality [13]. Retinal screening in remote areas has reported high image quality, attributed to the development of non-mydriatic fundus cameras. We evaluated the quality of non-mydriatic fundus photographs using a three-level scale, modified from the grading system of the FOTO-ED study [14]. Approximately 70% of the retinal images captured by a non-mydriatic fundus camera were graded as “readable” in our study, with 41.0% rated as “excellent” and 28.2% as “good”. This proportion was consistent with findings from other studies [15,16]. Lin et al. reported “excellent” (22.7%) and “good” (55.7%) image quality among patients without mydriasis, with 21.6% rated as “poor” [16]. Similarly, Maberley et al. [15] demonstrated “good” (25%) and “intermediate” (45%) image quality in non-mydriatic retinal photos, with 30% rated as “poor.” However, Yeh’s study reported over 95% of non-mydriatic fundus images as acceptable or of ideal quality [13], which is significantly higher than in our study. They explained that adding an eyecup to the camera optics helps achieve physiological mydriasis and reduces corneal reflections, thereby decreasing artifacts [13].

Upon comparing the diagnoses based on photos taken by a non-mydriatic camera in the infirmary with a second fundoscopic check in the hospital, 77.8% concordance was observed among the referral patients. This substantial agreement highlights the usefulness of the non-mydriatic images. However, it also underscores the limitations of the imaging equipment used. Some subtle retinal lesions outside the view of the fundus camera could only be detected with dilated indirect fundoscopy [13]. Additionally, certain macular lesions, such as diabetic macular edema and macular epiretinal membrane, were only revealed through OCT at the hospital. Discrepancies were often due to a lower threshold for disease screening, leading to initial over-diagnosis. Other factors, such as difficulties in obtaining readable images, also impacted accurate diagnoses and warrant further analysis as discussed herein.

Previous research has indicated that certain ocular conditions, such as cataracts, miosis, and vitreous hemorrhage, as well as factors such as age and race, may impact the quality of non-mydriatic fundoscopy images [11,12,14,17]. Our analysis identified the risk factors that adversely affect non-mydriatic fundus image quality, which include older age (*p* = 0.022), the presence of cataracts (*p* < 0.001), pseudophakia (*p* < 0.001), and diabetes mellitus (DM) (*p* < 0.001).

Several studies have shown that older age is associated with a decreased likelihood of obtaining high-quality fundus images [11,14,17,18,19]. More frequent occurrences of cataracts, a greater degree of miosis, and poorer cooperation were observed in older patients [11]. Lamirel et al. [14] also demonstrated that in emergency room settings, age was a significant predictor of image quality for non-mydriatic photographs. In some elderly patients, poor eye or head fixation and movement were noted during the taking of fundus photographs. Declining cognitive function and physical fitness also made it difficult for some elderly patients to follow instructions during the photography process. There was a higher rate of technical failure in fundus photography among older patients compared to younger ones.

Previous studies have suggested that diabetes mellitus is associated with poor image quality in non-mydriatic fundoscopy photographs. Scanlon PH et al. reported that the likelihood of obtaining ungradable fundus image quality increases by 2.6% for each additional year since a diabetes diagnosis, regardless of age [20]. In patients with diabetes, the pupillary sympathetic pathway is often impaired due to autonomic denervation of the pupil, resulting in a significantly smaller mean pupil size in dark-adapted conditions compared to controls [21]. This relatively constricted pupil state during fundus photography with a non-mydriatic camera leads to poorer quality retinal images compared to those from non-diabetic patients.

**Figure 5:**
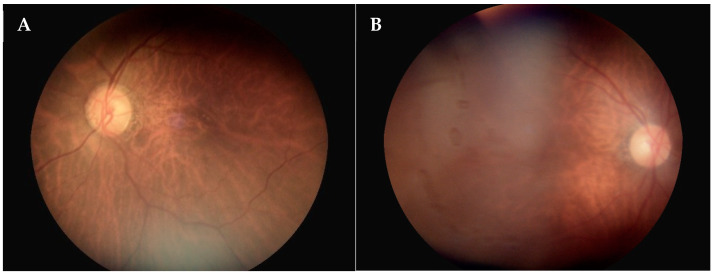
Examples of pseudophakic eyes showing decreased image quality. Fundus photographs showed inferior ghost images (**A**) and temporal light reflections (**B**). Both photos have white cellophane obscuring the retinal structures.

Lin et al. [16] reported that lens opacity was significantly associated with poor image quality. Cataracts, which lead to ocular media opacities, are linked to a decreased likelihood of obtaining acceptable retinal images, as observed in the current study. Davila et al. also noted that the presence of cataracts was associated with extremely poor retinal image gradability [17]. However, the previous literature did not discuss the association of pseudophakic status with the poor image quality of non-mydriatic fundoscopic photos. Many medical practitioners believe that transparent artificial lens implants will result in clearer fundoscopic images and expect the quality of retinal images to improve post-cataract surgery. Contrary to expectations, the presence of an intraocular lens (pseudophakia) was significantly associated with poor fundus image quality in our study. We hypothesize that reflections from the anterior surface of intraocular lenses may impair the image quality in non-mydriatic fundoscopy. Mooren et al. [22] validated a model to predict anterior IOL reflections. Intraocular lenses made from high refractive index materials, particularly those with a biconvex design, exhibit the highest reflectivity. The external surface reflections of an IOL are more than threefold higher than those from lenses with a lower refractive index, leading to the “cat’s eye” phenomenon observed in pseudophakic eyes by an outside observer [23]. This phenomenon reflects the flashlight of the non-mydriatic fundus camera and causes poor image quality in non-mydriatic fundoscopy photographs. Our study is pioneering in discovering that patients who have undergone cataract surgery with IOL implantation may experience decreased non-mydriatic retinal image quality. We found the fundus photos of pseudophakic patients exhibit more whitish cellophane, blocking the desirable structures (Figure 5). Conversely, the image quality of patients with intraocular lenses during mydriasis warrants further investigation.

In our study, we found no difference in the quality of fundus photos taken across various local infirmaries. Previous research demonstrated that the quality of fundus images captured with a non-mydriatic camera did not significantly vary among photographers with different levels of training [15]. Consequently, non-mydriatic fundoscopic photos are useful for diagnosing retinal diseases in teleophthalmology settings, provided that local infirmary staff receive appropriate training.

Our teleophthalmology services primarily focus on the image quality produced by our non-mydriatic fundoscopic camera, compared to other teleophthalmology studies. Additionally, we have identified the presence of intraocular lenses (pseudophakia) as a risk factor for poor image quality, a finding not previously reported in other studies.

We acknowledge that the current study has some limitations. For safety reasons, patients could not undergo mydriasis to compare fundus images before and after pupil dilation in the local infirmary setting. However, the majority of images were readable and allowed for accurate diagnosis. Regarding the risk factors affecting fundoscopy quality, we need to analyze more images for further validation.

## 5. Conclusions

In conclusion, our project demonstrated the feasibility of using a non-mydriatic digital fundus camera to detect various retinal disorders in teleophthalmology settings. The findings indicated that the non-mydriatic fundus camera could produce photographs with readable image quality, teaming up with the teleophthalmologic platform, resulting in substantial diagnostic concordance. Additionally, we identified pseudophakia as a unique risk factor that leads to poor fundoscopy image quality.

## Figures and Tables

**Table 1:** An overview of patients’ characteristics.

Variables	Male	Female	Total	*p*-Value
Number	210	419	629	
Age	65.67 ± 16.01	63.77 ± 16.12	64.40 ± 16.10	0.163
Age Group (*n*, %)				0.183
≦20 years	6 (2.9%)	10 (2.4%)	16 (2.5%)	
21–30 years	4 (1.9%)	9 (2.1%)	13 (2.1%)	
31–40 years	1 (0.5%)	21 (5.0%)	22 (3.5%)	
41–50 years	15 (7.1%)	34 (8.1%)	49 (7.8%)	
51–60 years	28 (13.3%)	63 (15.0%)	91 (14.5%)	
61–70 years	62 (29.5%)	114 (27.2%)	176 (28.0%)	
71–80 years	62 (29.5%)	117 (27.9%)	179 (28.5%)	
81–90 years	30 (14.3%)	50 (11.9%)	80 (12.7%)	
>90 years	2 (1.0%)	1 (0.2%)	3 (0.5%)	
Referral (*n*, %)	49 (23.3%)	77 (18.4%)	126 (20.0%)	0.143

Data are presented as *n* (%) or mean ± standard deviation. A *p*-value < 0.05 was considered statistically significant after test.

**Table 2:** Analysis of factors associated with poor-quality fundoscopy images (*n* = 1328).

Variables	Crude		Adjusted	
	OR (95% CI)	*p*-Value	OR (95% CI)	*p*-Value
Age	1.03 (1.02 to 1.04)	<0.001 *	1.02 (1.01 to 1.03)	0.004 *
Gender (M vs. F)	1.31 (1.02 to 1.68)	0.038 *	1.19 (0.90 to 1.57)	0.222
Location	-	-	-	-
Chishang	1.17 (0.83 to 1.65)	0.375	0.91 (0.62 to 1.33)	0.614
Haiduan	1.07 (0.76 to 1.50)	0.707	1.47 (0.99 to 2.18)	0.055
Luye	0.73 (0.52 to 1.02)	0.066	0.82 (0.56 to 1.19)	0.294
Yanping	0.87 (0.30 to 2.51)	0.795	3.03 (0.99 to 9.18)	0.051
Guanshan	Reference		Reference	
Pseudophakia	4.19 (2.98 to 5.90)	<0.001 *	4.06 (2.74 to 6.00)	<0.001 *
Cataract	2.19 (1.71 to 2.81)	<0.001 *	2.29 (1.69 to 3.11)	<0.001 *
Diabetes mellitus	3.86 (2.95 to 5.07)	<0.001 *	3.01 (2.25 to 4.01)	<0.001 *

Data are presented as odds ratios (95% CI). A * *p*-value < 0.05 was considered statistically significant after test.

## Data Availability

The data presented in this study are available on request from the corresponding author. The data are not publicly available due to patients’ confidentiality.
